# Characterization and Discrimination of Chinese Marinated Pork Hocks by Volatile Compound Profiling Using Solid Phase Microextraction Gas Chromatography-Mass Spectrometry/Olfactometry, Electronic Nose and Chemometrics

**DOI:** 10.3390/molecules24071385

**Published:** 2019-04-09

**Authors:** Dong Han, Si Mi, Chun-Hui Zhang, Juan Li, Huan-Lu Song, Marie-Laure Fauconnier, Eva Tyteca

**Affiliations:** 1 Institute of Food Science and Technology, Chinese Academy of Agricultural Sciences, Beijing 100193, China; orange_1101@126.com (D.H.); misi@caas.cn (S.M.); 15711388983@163.com (J.L.); 2 Gembloux Agro-bio Technology, University of Liege, 25030 Gembloux, Belgium; marie-laure.fauconnier@ulg.ac.be (M.-L.F.); eva.tyteca@gmail.com (E.T.); 3 Laboratory of Molecular Sensory Science, Beijing Technology and Business University, Beijing 100048, China; songhl@th.btbu.edu.cn

**Keywords:** characterization, discrimination, Chinese marinated pork hocks, odour activity value, key odour-active compounds, potential flavour markers

## Abstract

The primary aim of this study was to investigate volatile constituents for the differentiation of Chinese marinated pork hocks from four local brands, Dahongmen (DHM), Daoxiangcun (DXC), Henghuitong (HHT) and Tianfuhao (TFH). To this end the volatile constituents were evaluated by gas chromatography-mass spectrometry/olfactometry (GC-MS/O), electronic nose (E-nose) and chemometrics. A total of 62 volatile compounds were identified and quantified in all pork hocks, and 24 of them were considered as odour-active compounds because their odour activity values (OAVs) were greater than 1. Hexanal (OAV at 3.6–20.3), octanal (OAV at 30.3–47.5), nonanal (OAV at 68.6–166.3), 1,8-cineole (OAV at 36.4–133.3), anethole (OAV at 5.9–28.3) and 2-pentylfuran (OAV at 3.5–29.7) were the key odour-active compounds contributing to the integral flavour of the marinated pork hocks. According to principal component analysis (PCA) and partial least squares-discriminant analysis (PLS-DA) of GC-MS/O and E-nose data, the results showed that the marinated pork hocks were clearly separated into three groups: DHM, HHT, and DXC-TFH. Nine odour-active compounds, heptanal, nonanal, 3-carene, d-limonene, β-phellandrene, *p*-cymene, eugenol, 2-ethylfuran and 2-pentylfuran, were determined to represent potential flavour markers for the discrimination of marinated pork hocks. This study indicated the feasibility of using GC-MS/O coupled with the E-nose method for the differentiation of the volatile profile in different brands of marinated pork hocks.

## 1. Introduction

The preparation of marinated pork hocks in China can be dated back to thousands of years ago. The traditional marinating procedure is as follows: the fresh meat is immersed in marinade for a certain period of time after meat is cooked in liquid at 98 ± 2 °C for 1 h or longer [1]. Marinated pork hock, a part of the Chinese time-honoured and intangible cultural heritage, is appreciated by Chinese consumers due to its distinct sensory characteristics, such as tender texture, bright colour and rich flavour. Among all the sensory attributes, flavour has been rated as one of the most important attributes for this type of products. To date, more than 1000 odorous compounds have been identified in various meat products, including aldehydes, ketones, alcohols, esters, heterocyclic compounds and sulfur-containing compounds [2]. The comparison of volatiles in the marinated drumsticks using the traditional and quantitative marinated methods found 44 and 60 volatile flavour compounds present in these two types of products, respectively, which showed that the processing method can affect the flavour composition of marinated products greatly [3]. A total of 149 volatile compounds that have been identified in dry-cured hams exposed to different processing methods, and most abundance volatiles in the ham samples were aldehydes [4]. Volatile flavour constituents in roasted pork from miniature pigs were studied, in which aldehydes are believed to play an important role in the flavour composition [5]. All of these studies above mainly focused on the qualitative and quantitative analysis of the volatile compounds from the different meat products, such as dry-cured hams [4,6], roasted pork [5] and braised chicken [3,7]. However, few studies have been reported on the flavour profiling of Chinese marinated pork hocks.

Solid phase microextraction and gas chromatography-mass spectrometry (SPME-GC-MS) have been applied as a prevalent analytical technique for food aroma analysis due to its ability to separate and identify volatile compounds [8,9,10]. However, it is difficult to distinguish the contribution of each single volatile to the integral food aroma profile. The odour activity value (OAV) is thus introduced. OAV can be calculated based on dividing the concentration of a compound by its mean recognition threshold in the matrix [11,12]. Moreover, one of the weaknesses of the GC-MS method is that it cannot associate compounds with the actual sensory experience. Therefore, linking the sensory experience and chemical compounds identified by gas chromatography-olfactometry (GC-O) was attempted. The most important aroma-active compounds of Beijing roast duck were identified by using aroma extract dilution analysis (AEDA), dynamic headspace dilution analysis, GC-MS/O and OAVs [13]. 2,3-Butanedione, 2,5-dimethylpyrazine, 3-methylbutanal, 2-acetyl-1-pyrroline and 2-acetylthiazole were the key potent contributors in steamed mangrove crab, as determined on the basis of GC-MS/O methods [14]. The volatile compounds were extracted by the simultaneous distillation extraction (SDE) and 31 odour-active compounds were detected and identified in cooked meat of farmed obscure puffer by GC-MS/O [15]. The odorous sulphur compounds and three furans were considered as the responsibilities for the “meaty, cooked ham” notes in cooked ham [16]. These relevant research projects were about the flavour analysis of roast, steamed and cooked meat. However, a comprehensive GC-MS/O method for the identification and quantification of the volatile compounds in the marinated pork hocks has never been reported.

Nowadays, some studies have been reported on characterization and classification of different food using chemometrics analysis. The selected China’s domestic pork were characterized and discriminated using an LC-MS-based lipidomics analysis [17]. The ICP-MS and multivariate analysis of the mineral elementals were applied for the authentication of Taihe black-boned silky fowl muscles [18]. The volatile flavour composition can be also used as discriminating parameter for identifying the cooked pork samples from four pig varieties [19]. However, none of these studies regarding the discrimination of the marinated pork hocks has been used for chemometrics analysis of the volatile compounds. This study was aimed to investigate the volatile profiles of marinated pork hocks from different brands and to determine the key odour-active compounds and potential flavour markers using GC-MS/O. The multivariate statistical techniques, such as PCA and PLS-DA were used to understand the similarities and differences between the marinated pork hocks from different brands.

## 2. Results and Discussion

### 2.1. Volatile Profile of Marinated Pork Hocks Characterized by GC-MS/O

#### 2.1.1. Volatile Composition of Marinated Pork Hocks

The odour descriptions, thresholds and relative concentrations of the volatile compounds identified in the marinated pork hocks are presented in Table 1. A total of 62 volatile compounds were detected in the marinated pork hocks, and there were 35, 37, 33 and 25 volatile compounds in DHM, DXC, HHT and TFH, respectively. These compounds can be further categorized into 10 major groups, as follows: 13 aldehydes, nine alcohols, five ketones, three esters, 14 hydrocarbons, two ethers, two phenols, seven furans, three *N*-containing compounds and four *S*-containing compounds. Total estimated concentrations of volatile compounds in DHM, DXC, HHT and TFH were 1224.2 μg·kg^−1^, 1073.4 μg·kg^−1^, 1791.8 μg·kg^−1^ and 822.7 μg·kg^−1^, respectively. TFH contained the least number and lowest levels of volatile constituents. Conversely, the greatest number and highest levels of volatile compositions were detected in DXC and HHT, respectively.

The majority of aldehydes significantly contributed to the flavour profiles of various food matrices due to their low odour thresholds [11,20]. Aldehydes are mainly generated from two pathways, i.e., lipid oxidation and Strecker degradation of amino acids [3,5]. In this study, the 2,4-decadienal isomer with fatty and deep-fried odour had the lowest threshold of 0.07 μg·kg^−1^ in DHM. However, this compound was not observed in the other three pork hock samples. Iu et al. [21] reported that the 2,4-decadienal isomer was considered as an oxidation product of linoleic acid, which was the main polyunsaturated fatty acid found in cooked cured pork ham. Nonanal with a citrus and fatty odour was identified and had the highest concentration in all samples. This aldehyde was generated from lipid oxidative degradation [6]. Additionally, the remaining four aldehyde compounds, including hexanal, octanal and benzaldehyde, were also detected in all samples. Among these compounds, benzaldehyde (with a bitter almond smell) has the larger odour threshold, which demonstrates the lesser contributions to the aroma profiles of the marinated pork hocks. Note that the aroma profile of DXC was characterized by the presence of 2-methylbutanal and 3-methylbutanal with a nutty odour, which were not detected in the other three samples. Gu et al. [11] reported that 2-methylbutanal was known to be a Strecker reaction product of isoleucine in steamed Chinese mitten crab. Liu et al. [22] found that 3-methylbutanal was formed in salty boiled duck in water, and the formation may be associated with leucine.

Tanchotikul and Hsieh [23] reported that alcohols were one of the key flavour compounds in steamed Rangia clam, which could be associated with the decomposition of hydroperoxides of fatty acids or the reduction of aldehydes. Regarding the relative concentrations of alcohols detected in the marinated pork hocks, 1,8-cineole, linalool and terpinen-4-ol were detected in all samples. As shown in Table 1, the relative concentration of 1,8-cineole with the minimum threshold was significantly higher than all other listed alcohol compounds, which indicated its greatest contribution to the complete flavour profile of marinated pork samples. In addition, linalool and 1-octen-3-ol were known to be the most important aroma-active alcohols and have been found in the essential oil [24] and fish products [12], respectively. The β-oxidation of linoleic acid has been considered as the main pathway to form 1-octen-3-ol in dry cured loin [25]. The odour threshold of 1-octen-3-ol (2 μg·kg^−1^) was three times lower than that of linalool (6 μg·kg^−1^); therefore, a higher OAV was achieved by 1-octen-3-ol in DHM, HHT and TFH compared with linalool. In contrast to the above discussed volatile alcohols, terpinen-4-ol, α-terpineol and 2-phenylethanol had very high thresholds, which indicated that they were not the main flavour substances but exerted a synergistic influence on the total flavour.

As for ether compounds, estragole and anethol were identified in all marinated pork hocks (Table 1). The relative concentrations of estragole and anethol in DHM and TFH were significantly higher than in DXC and HHT (*p* < 0.05), which could be explained by the greater quantities of herbs and spices used in the processing of DHM and TFH. Yao et al. [26] pointed out that both compounds are the main components in aniseed plants.

Another main observation from Table 1 is the relatively high odour threshold of hydrocarbons, which results in low contributions for the majority of the hydrocarbons except ethyl acetate, 3-carene, d-limonene and β-phellandrene. As reported, hydrocarbons can be derived from alkyl radicals via lipid auto-oxidation processes [29]. Other types of volatiles including ketones, esters and phenols are also considered as a flavour auxiliary of the marinated pork hocks, although they have relatively high thresholds.

Furans refer to a group of heterocyclic compounds that were structurally characterized with the oxygen atom in the ring. Giri et al. [20] reported that furans might be derived from the dehydration of carbohydrates or the Amadori rearrangement procedure. Taylor and Mottram [30] suggested that the oxidation of fatty acids could be another pathway for the formation of furans. Among six furans listed in Table 1, 2-pentylfuran with the lower threshold (6.0 μg·kg^−1^) had the highest relative concentration in all samples, and 2-ethylfuran with the lowest threshold (2.3 μg·kg^−1^) had the highest relative concentration in only HHT. These two furans were oxidative degradation products of linolenate [11,13] and were considered as the most important flavour compounds contributing to meat products [11].

Several *N*- and *S*-containing compounds were detected in the marinated pork hocks. They were mainly derived from the catabolism of proteins, free amino acids and nucleic acids [13]. The 2-acetylthiazole with roasted and caramel notes was usually considered as an important flavour compound contributing to the flavour of cooked meat [13,31].

#### 2.1.2. OAVs of the Odour-Active Compounds

The OAV (Equation (3)) was employed to evaluate the contribution of volatile compounds to the aroma profile of the investigated samples. The results are summarized in Table 2. 

Twenty-four volatile compounds with OAVs > 1 were selected as odour-active compounds contributing primarily to the total flavour of the marinated pork hocks. A point worth emphasizing is that six odour-active components with relatively high OAVs were simultaneously detected in four samples: hexanal (OAV at 3.6–20.3), octanal (OAV at 30.3–47.5), nonanal (OAV at 68.6–166.3), 1,8-cineole (OAV at 36.4–133.3), anethol (OAV at 5.9–28.3) and 2-pentylfuran (OAV at 3.5–29.7). 1,8-Cineole and linalool constituted a large portion of the specific aroma of Chinese marinated meat products and could have originated from the Chinese traditional spices. Hexanal, octanal, nonanal and 2-pentylfuran with fat and meat flavours were generated from the boiling procedure of the marinated meat products. These components were defined as the key odour-active compounds due to their significant contributions (*p* < 0.05) to the integral flavour. Considering the VIP scores and *p*-values of odour active compounds, nine of the compounds had a VIP score > 1 and *p*-value < 0.05 and were considered as potential discriminatory markers for the differentiation of marinated pork hocks. These odour-active compounds included heptanal, nonanal, 3-carene, d-limonene, β-phellandrene, *p*-cymene, eugenol, 2-ethylfuran and 2-pentylfuran.

### 2.2. Discrimination of Marinated Pork Hocks by GC-MS/O

**Figure 1:**
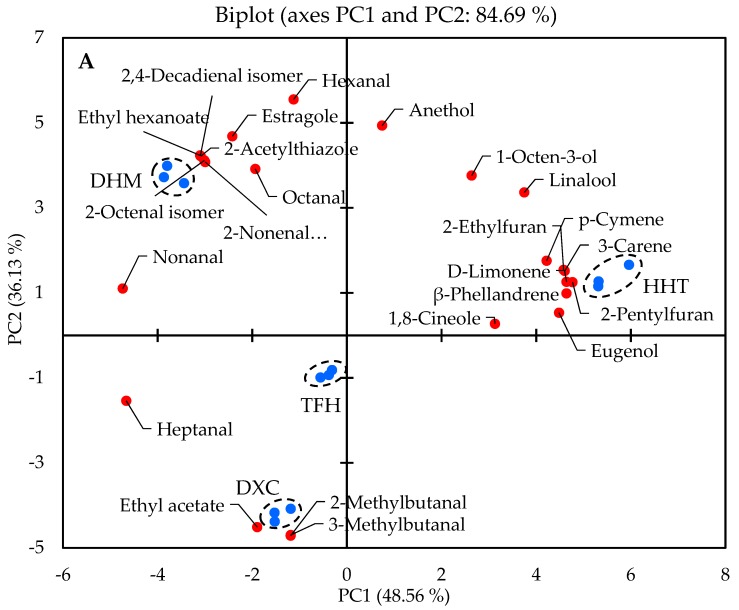

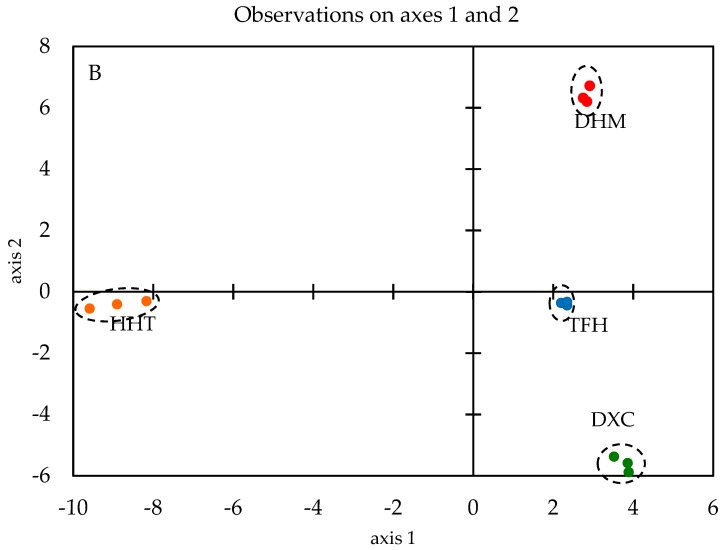
(**A**) PCA for odour-active compounds of the four marinated pork hocks. The blue dots represent the samples from marinated pork hocks, and the red dots represent odour-active compounds. (**B**) PLS-DA score plot from different marinated samples (R^2^X = 0.978, R^2^Y = 0.997, Q^2^ = 0.994). The red dots represent DHM, the yellow dots represent HHT, the blue dots represent TFH, and the green dots represent DXC.

To visualize a total picture of the distributions of 24 odour-active compounds (OAV > 1) in all samples, PCA was applied (Figure 1). The two principal axes accounted for 84.69% of the entire variations of the four pork hocks; the two PCA components, PC1 and PC2, explained 48.56% and 36.13% of the variation, respectively. As shown in Figure 1A, it can be found that the sample dots of the marinated pork hocks were well separated. PC1 clearly distinguished DHM and HHT. DHM was in the positive side of PC1, while HHT appeared in the negative side, indicating that there were obvious differences of flavour features. Both DXC and TFH were located in the lower left quadrant of PC and close to each other, which meant that they have similar flavour. Hence, the four different marinated pork hocks were divided into three groups, i.e., group I: DHM, group II: HHT, and group III: DXC-TFH.

Moreover, the major odour-active compounds contributing to DHM were hexanal (green, grass), 2,4-decadienal isomer (fatty, deep-fried), 2-acetylthiazole (caramel, sweaty), ethyl hexanoate (fruity, sweet), 2-nonenal isomer (fatty, cucumber), 2-octenal isomer (fatty, green), estragole (aniseed-like) and octanal (orange peel, fatty). As seen from Table 2, the OAV values of these aldehyde compounds in DHM were significantly higher than those in the other samples (*p <* 0.05). As had been reported on all types of foodstuff [6,32,33], the eight compounds above have been widely studied with respect to their sources and contributions to food aroma [4,34,35]. HHT was in the first quadrant and highly associated with four hydrocarbons (*p*-cymene, 3-carene, d-limonene and β-phellandrene) and two furans (2-ethylfuran and 2-pentylfuran). Among them, the hydrocarbon compounds could originate from the animal feeds [24] and Chinese traditional spices [3]. 2-ethylfuran, 2-pentylfuran and eugenol could have been found in the cooked meat products [28,36] and the traditional salted vegetables [27]. Only three volatile components were related to DXC and TFH, including ethyl acetate, 2-methylbutanal and 3-methylbutanal. As discussed previously, the formation of 2-methylbutanal and 3-methylbutanal can be attributed to the amino acid Strecker reaction [6,20].

In addition to PCA analysis, PLS-DA was applied for the discrimination of all marinated pork hocks. Figure 1B illustrated the PLS-DA score of the marinated samples. HHT was located on the negative side of axis 1, DHM was found on the positive side of axis 2, and TFH and DXC were clustered on the negative side of axis 2. Hence, there were three separate groups (HHT, DHM and DXC-TFH) for marinated pork hocks. It could also be concluded that the flavour of DXC and TFH was similar, and they were different from HHT and DHM.

### 2.3. Volatile Profile of Marinated Pork Hocks Characterized by E-Nose

**Figure 2:**
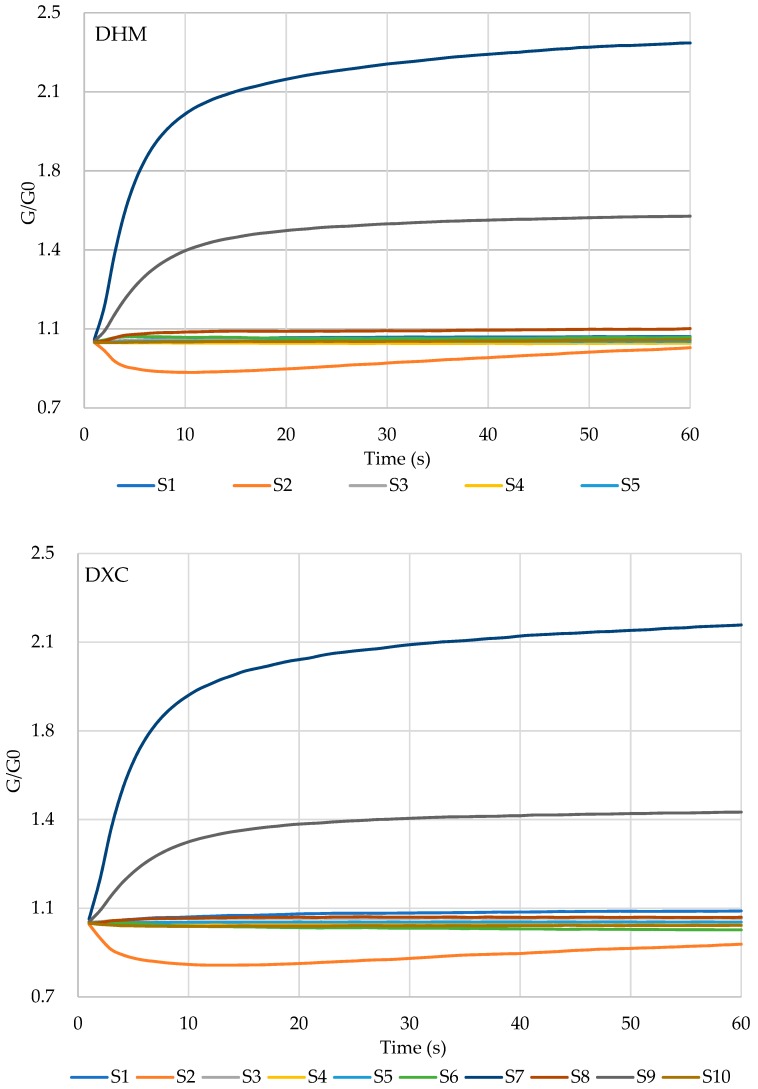

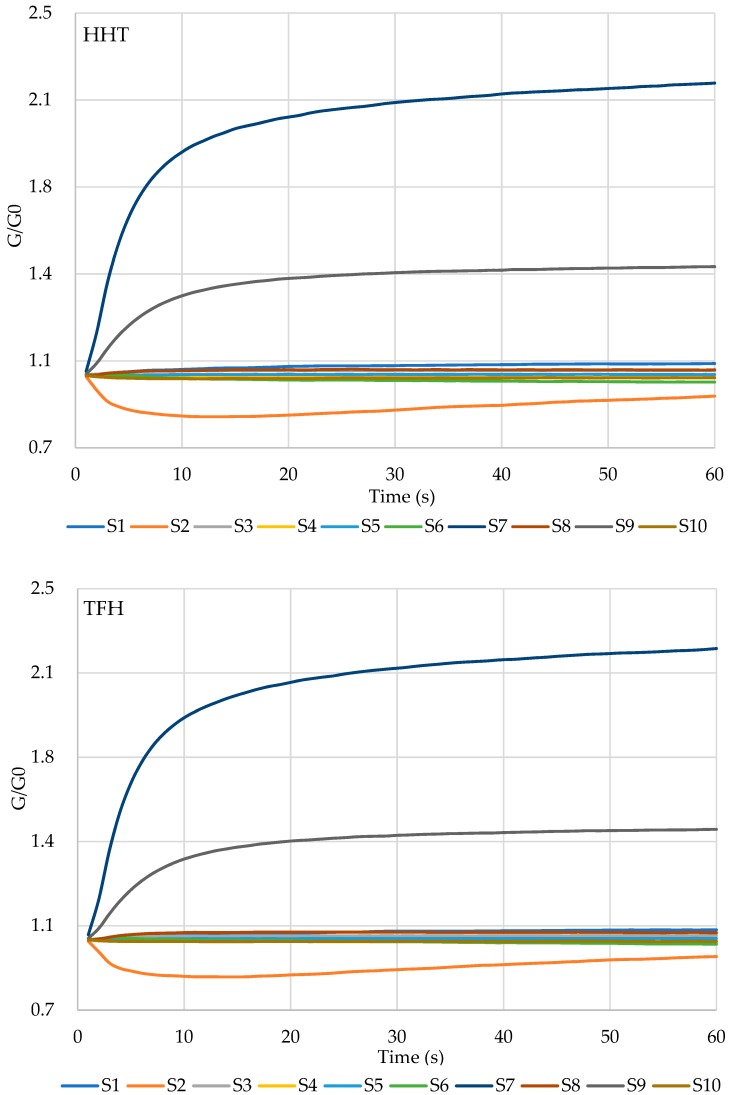
Response curves of E-nose sensors (S1–S10) to DHM, DXC, HHT and TFH.

The E-nose responses to the marinated pork hocks were shown in Figure 2, where G/G0 was considered as the response value. 

Each curve represented the response values of the corresponding sensors varying with time. The variation trends of signals in all samples showed similar changes. The response values of S2 (broad range of nitrous oxides), S7 (terpenes and sulphur-containing organic compounds), and S9 (aromatics and organic sulfides) obviously increased, whereas those of S1 (aromatic compounds) and S8 (broad alcohols) gradually increase to a slight extent. Meanwhile, the signals of S3 (aroma components, ammonia) and S5 (alkane, aromatics, and small polar compounds) showed insignificant changes. The values of G/G0 of S4, S6 and S10 were below one. These sensors were mainly sensitive to hydrogen, broad methane and aliphatic methane, respectively. Compared with all marinated samples, it was found that the signal intensities of DHM showed apparent differences. This result suggested different flavour characteristics. According to the sensor signals, it was difficult to distinguish the marinated samples. Therefore, further analysis was applied by PCA and PLS-DA in following study.

### 2.4. Discrimination of Marinated Pork Hocks by E-Nose

**Figure 3:**
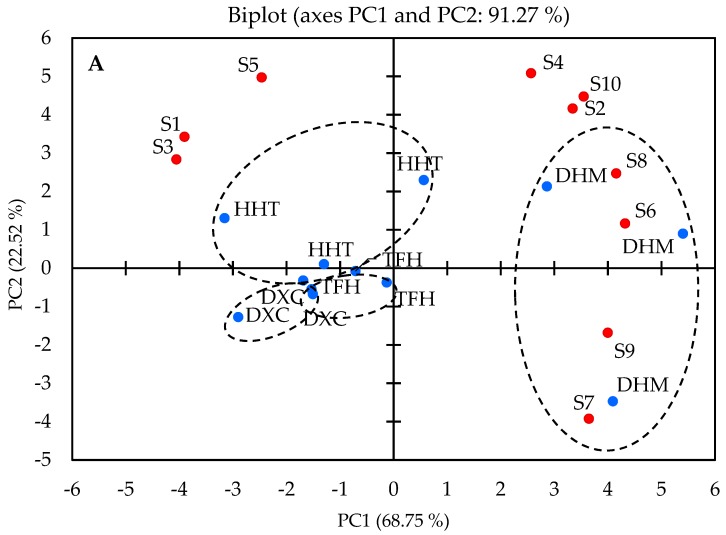

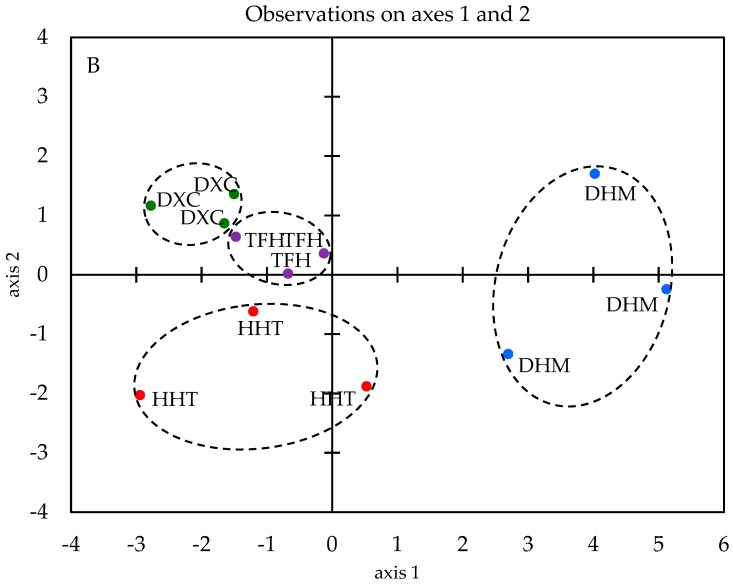
(**A**) Biplot (score plots and load plots) for PCA based on sensor response data. The blue dots represent the samples from marinated pork hocks, and the red dots represent different sensors. (**B**) PLS-DA of E-nose response data for different marinated samples (R^2^X = 0.997, R^2^Y = 0.829, Q^2^ = 0.407). The blue dots represent DHM, the red dots represent HHT, the purple dots represent TFH, and the green dots represent DXC.

E-nose analysis was performed to evaluate the differences in the aroma profiles of four brands of the marinated pork hocks. The PCA score and load plots of the data obtained by E-nose are shown in Figure 3A. The plot consists of two axes, PC1 and PC2. PC1 explains 68.75%, whereas PC2 explains 22.52% of the sample variance. The total cumulative contribution rate of PC1 and PC2 exceeded 85.0% [37], which indicated that the maximum variation of the aroma compositions of the marinated pork hocks was well explained by PCA analysis [34].

Apart from PCA analysis, PLS-DA was used to distinguish all marinated pork hocks. From Figure 3B, the data points of DXC and TFH were closely allocated in the second quadrant; the data points of DHM were on the positive side in axis 1 and those of HHT were in the third quadrant and on the negative side of axis 1. This means that DXC and TFH share similar flavour profiles, although there are numerous different compounds (aldehydes and hydrocarbons) for DHM and HHT.

### 2.5. Sensory Analysis of Marinated Pork Hocks

**Figure 4:**
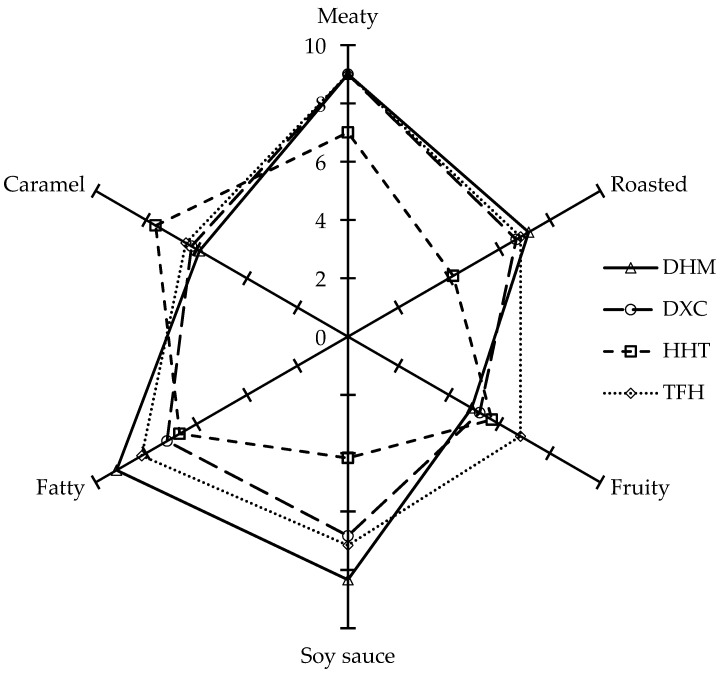
Radar charts of sensory analysis of the marinated pork hocks.

The sensory descriptive analysis of the flavour profiles of each sample is shown in Figure 4. The aroma of the marinated pork hocks was described as having a meaty odour, roasted odour, fruity odour, soy sauce odour, fatty odour and caramel notes. The intensities of fatty odour, meaty odour, roasted odour and soy sauce odour in DHM were higher than those in other samples, which could be mainly attributed to aldehydes and *N*- and *S*-containing compounds (e.g., nonanal, 2-nonenal isomer, 2,4-decadienal isomer, 2-acetylthiazole and 3-(methylthio)propanol). It was shown that this result was consistent with Table 1. These volatile compounds were detected in the pork broth of black pig [32] and Chinese-type soy sauce [38]. The strong fruity smell was presented in TFH because of the high levels of contributions of total alcohols (24.7%). Wang et al. [6] reported that alcohols have pleasant fruity and floral odours in Chinese dry-cured ham. In addition, caramel notes of HHT had the highest scores in all samples, indicating that HHT was highly associated with furans, such as 2-ethylfuran and 2-pentylfuran.

## 3. Materials and Methods

### 3.1. Materials and Chemicals

The study procedures were approved by the Animal Care and Use Committee of the Institute of Food Science and Technology, Chinese Academy of Agricultural Sciences, and were performed in accordance with animal welfare and ethics. Twelve commercial marinated pork hocks were purchased, including the following Chinese brands: Beijing Ershang Group Dahongmen Meat Food Co. Ltd. (labelled as DHM, Beijing, China), Beijing Daoxiangcun Food Co. Ltd. (labelled as DXC, Beijing, China), Beijing Henghuitong Meat Food Co. Ltd. (labelled as HHT, Beijing, China), and Beijing Tianfuhao Food Co. Ltd. (labelled as TFH, Beijing, China). For each brand, three different lot numbers were collected. According to the tracking information, the products were processed using Duroc × (Yorkshire × Landrace) pigs (*n* =12, aged 5–6 months and with body weights of 90–95 kg), and all of the pigs were slaughtered following routine abattoir procedures. After chilling at 2–4 °C for 24 h, the pork hocks were dissected from the individual carcasses. To produce marinated pork hocks, hocks were first boiled in water at 100 °C for 10 min to remove blood and then transferred to special marinades for 45 min at 100 °C. The marinade information for each product was summarized in Table 3. The meat was trimmed to the skin, visible external fat and connective tissues. To minimize the deterioration of volatile components, all samples were cut into small pieces (5 mm × 5 mm × 5 mm) and stored at −18 °C until needed.

Saturated alkanes C_7_–C_30_ (1000 μg/mL for each component in hexane) and 2-methyl-3-heptanone (99%) were obtained from Sigma-Aldrich (Shanghai, China).

### 3.2. Solid Phase Microextraction of Volatile Compounds

The SPME manual device (Supelco, Inc., Bellefonte, PA, USA) equipped with a 50/30 μm divinylbenzene/carboxen/polydimethylsiloxane (DVB/CAR/PDMS) fibre was employed for the extraction of volatile compounds from the marinated pork hocks. The extraction was performed according to the method proposed by Yao et al. [26] with some modifications. Briefly, 5.0 g of meat sample was accurately weighed and transferred to a 40 mL headspace flask. Then, 1 μL of 2-methyl-3-heptanone solution at 0.41 mg/mL (dissolved in hexane) was added to the homogenized meat sample, acting as the internal standard before the SPME processing. The sample was equilibrated at 60 °C for 20 min and extracted with the selected fibre for 40 min at the same temperature. Upon completion, the fibre was inserted into the injection port (250 °C) of the GC instrument to desorb the analytes for 5 min. All samples were extracted in triplicate.

### 3.3. GC-MS/O Analysis of Volatile Compounds

All volatile compounds were analysed by GC-MS using an Agilent 7890A gas chromatograph and an Agilent Model 7000B series mass-selective detector with a quadrupole mass analyser (Agilent Technologies, Inc., Santa Clara, CA, USA). An olfactory detector port (Sniffer 9000; Brechbuhler, Schlieren, Switzerland) was coupled with the GC/MS system. After samples were injected, two types of capillary columns, polar DB-Wax and non-polar DB-5 (30 m × 0.32 mm × 0.25 µm; J & W Scientific, Inc., Folsom, CA, USA), were used to perform the separation. Ultra-high purity helium (≥99.999%) was employed as the carrier gas with a constant flow rate of 1.2 mL/min. The oven programme was as follows: the initial column temperature was maintained at 40 °C for 3 min, then increased to 200 °C at a rate of 5 °C/min, and finally increased to 230 °C (DB-Wax) and 250 °C (DB-5) at 10 °C/min for 3 min. The transfer line temperatures were 240 °C (DB-Wax) and 270 °C (DB-5). The effluent from the capillary column was split between the mass spectrometry detector and the olfactory detector port at a ratio of 5:1 (*v/v*). The working conditions of MS were set as follows: ionization energy at 70 eV; scan range at 50–400 *m/z*; and ion source temperature at 230 °C. For the GC-O analysis, the occurrence time and characteristics were recorded by six experienced panellists during the sniffing test. Humidified air was supplied to the sniff port with a flow of 30 mL/min to avoid dryness of the nasal mucosa.

### 3.4. Identification and Quantification of Volatile Compounds

The volatile components were identified by comparing their electron ionization (EI) spectra with the database records provided by the National Institute of Standards and Technology (NIST) Mass Spectral Library (Version 2.0, Gaithersburg, MD, USA). The qualitative determination of these volatile compounds was further confirmed via matching their linear retention indices (LRIs) and odour descriptions in the literature and online databases (http://www.flavornet.org; http://www.odour.org.uk). The LRI values were computed according to the following equation [39]:

(1) $$\mathrm{LRI}=100\times \left(\frac{\mathrm{Rt}\;\left(i\right)-\mathrm{Rt}\left(n\right)}{\mathrm{Rt}\left(n+1\right)-\mathrm{Rt}\left(n\right)+n}\right)$$

where Rt (i) is the retention time of the individual compound under investigation (i) and Rt (n) and Rt (n + 1) refer to the retention times of *n*-alkanes that elute before and after the target compound (i) for the same chromatographic conditions.

Quantitative analysis of the identified volatile compounds was achieved by adding 2-methyl-3-heptanone (internal standard, IS) to the samples prior to the SPME procedures. The relative concentrations of the volatile constituents were determined by the GC-peak areas of calibration curves and the ratios of the target analytes relative to 2-methyl-3-heptanone. The final results were expressed as µg volatile compounds/kg of the marinated pork hocks. Each value represented the average of triplicate determinations. 2-Methyl-3-heptanone was used as the internal standard without considering the calibration factors; thus, all calibration factors were considered to be 1.00. The equation is written as follows:

(2) Conc(μgkg)=Peak area ratio(volatileIS)×0.41μg(IS)5 g(pork hock sample) × 1000

The OAV is known as the ratio of the relative concentration (C*_i_*), which is the value of the identified compounds, to the odour threshold (OT) in water. The OAV can be calculated by the following additional equation:

(3) $${\mathrm{OAV}}_{i}=\frac{C_{i}}{{\mathrm{OT}}_{i}}$$

Compounds with OAV > 1 were considered as odour-active compounds [12].

### 3.5. E-Nose Analysis of Marinated Pork Hocks

A portable electronic nose (PEN3) with an enrichment and desorption unit (EDU) from Win Muster Airsense Analytics, Inc. (Airsense, Schwerin, Germany), was employed to investigate the odour profiles of pork hock samples. The PEN3 is composed of a sampling apparatus, a detector unit containing ten metal oxide sensors [38], and pattern identification software for data recording and elaboration [40]. Approximately 1.00 g of the marinated pork hocks was added to a 10 mL glass vial. Filtered and dried air with flow rate of 300 mL/min was employed for the headspace injection. The data acquisition period was 60 s, and an additional 180 s was required for system rebalancing. All samples had three replicates and were measured under the same conditions.

### 3.6. Sensory Evaluation of Marinated Pork Hocks

The panel for sensory evaluation included eight trained panellists (four males and four females, aged 25–35 years) from the Chinese Academy of Agriculture, Beijing. All assessors offered at least one year of experience in the descriptive analysis of marinated meat products. 

To train the sensory panel to be familiar with the sensory characteristics of marinated pork hocks, training sessions were conducted for 12 weeks (2 times per week), and each session took approximately 2 h. During the training sessions, the panellists, on the basis of available literature [41,42], developed and defined the sensory attributes, reference standard samples and their intensities (Table 4). Different marinated pork hock samples were coded with three-digit randomized numbers and served in random order to prevent bias. The evaluation was performed at room temperature, one time each, and with a 5 min wait between samples. The panellists were asked to evaluate six sensory attributes, namely, fatty odour, meaty odour, caramel odour, soy sauce odour, fruity odour and roasted odour. The intensities of six descriptive sensory attributes were evaluated using a 10 cm unstructured line [43] ranging from “not perceivable” to “strongly perceivable.” The data were presented as the mean values of scores of each odour note and plotted in the radar charts.

### 3.7. Statistical Analysis

Contents of volatile compounds were presented as the mean ± standard deviation (SD). One-way analysis of variance (ANOVA) and Duncan’s multiple range tests were carried out by using SPSS software (v. 19.0, SPSS, Inc., Chicago, IL, USA). The significance level was set at *p* < 0.05. PCA and PLS-DA were performed based on the mean OAV of odour-active compounds (OAV > 1) using the software XLSTAT (2016) from Addinsoft (Barcelona, Spain). The odour-active compounds with variable importance, indicated by the projection (VIP) score of > 1 in the PLS-DA analysis and p-value of < 0.05, were considered as significant differences among all marinated pork hocks. Likewise, PCA and PLS-DA of E-nose data were also conducted with XLSTAT (Addinsoft Inc, Longlsland, NY, USA, 2016). All experiments were performed in triplicate.

## 4. Conclusions

In this study, 62 volatile compounds were identified and quantified in marinated pork hocks using SPME-GC-MS/O. These compounds can be divided into ten categories, including aldehydes, alcohols, ketones, esters, hydrocarbons, ethers, phenols, furans, *N*-containing compounds and *S*-containing compounds. The key odour-active volatiles of all evaluated samples were determined as hexanal, octanal, nonanal, 1,8-cineole, anethol and 2-pentylfuran due to their relatively higher OAVs compared with other compounds. Moreover, through multivariate statistics including PCA and PLS-DA analysis, marinated pork hocks of four brands could be classified into three separate groups (DHM, HHT and DXC-TFH). Nine odour-active compounds were determined as potential flavour makers for the differentiation of marinated pork hocks. These analyses provided a reliable method to determine and distinguish the volatile profiles of different varieties of samples using GC-MS/O and E-nose.

## Figures and Tables

**Table 1:** Odour descriptions, odour thresholds and relative concentrations of volatile compounds in marinated pork hocks by GC-MS/O.

Code	Compound	Formula	DB-wax ^e^	DB-5 ^f^	Identification	^g^ Odour Descriptions	^h^ Odour Threshold (μg·kg^−1^)	Relative Concentration (μg·kg^−1^)			
								DHM	DXC	HHT	TFH
	Aldehydes (13)							372.0 ± 9.2a	322.1 ± 18.7a	142.5 ± 10.1b	377.0 ± 3.8a
1	2-Methylbutanal	C_5_H_10_O	902	680	MS, LRI, O	Nutty	1	^j^ N.D.	10.0 ± 1.2	N.D.	N.D.
2	3-Methylbutanal	C_5_H_10_O	906	^i^ N.A.	MS, LRI, O	Almond, nutty	1.1	N.D.	11.7 ± 1.8	N.D.	N.D.
3	Hexanal	C_6_H_12_O	1073	800	MS, LRI, O	Green, grass,	4	81.0 ± 3.5a	14.4 ± 1.6d	46.9 ± 4.6b	35.6 ± 1.2c
4	Heptanal	C_7_H_14_O	1179	901	MS, LRI, O	Fatty, oily	3	23.9 ± 1.5a	25.5 ± 3.1a	N.D.	16.4 ± 2.3b
5	Octanal	C_8_H_16_O	1284	1003	MS, LRI, O	Orange peel	0.7	33.2 ± 2.3a	21.2 ± 1.8c	25.1 ± 2.2b	31.9 ± 0.2a
6	Nonanal	C_9_H_18_O	1389	1104	MS, LRI, O	Citrus, fatty	1	166.6 ± 4.6a	122.4 ± 9.5c	68.6 ± 4.9d	135.9 ± 3.2b
7	2-Octenal isomer	C_8_H_14_O	1426	N.A.	MS, LRI, O	Fatty, green	3	3.7 ± 1.1	N.D.	N.D.	N.D.
8	Benzaldehyde	C_7_H_6_O	1520	961	MS, LRI, O	Bitter almond	350	29.2 ± 1.8c	102.9 ± 1.3b	N.D.	156.2 ± 1.7a
9	2-Nonenal isomer	C_9_H_16_O	1533	N.A.	MS, LRI, O	Fatty, cucumber	0.19	4.2 ± 1.1	N.D.	N.D.	N.D.
10	Anisaldehyde	C_8_H_8_O_2_	1683	N.A.	MS, LRI, O	Mint, sweet	27	13.8 ± 2.1a	12.7 ± 2.0a	N.D.	N.D.
11	2,4-Decadienal isomer	C_10_H_16_O	1719	N.A.	MS, LRI, O	Fatty, deep-fried	0.07	15.8 ± 1.2	N.D.	N.D.	N.D.
12	Pentadecanal	C_15_H_30_O	N.A.	1712	MS, LRI	Fresh	N.A.	N.D.	0.2 ± 0.1	N.D.	N.D.
13	Hexadecanal	C_16_H_30_O	N.A.	1793	MS, LRI	Cardboard	N.A.	0.5 ± 0.1c	1.1 ± 0.1b	1.8 ± 0.3a	1.1 ± 0.3b
	Alcohols (9)							127.4 ± 0.6c	107.5 ± 7.4d	249.8 ± 13.4a	202.8 ± 15.2b
14	1,8-Cineole	C_10_H_18_O	1204	1034	MS, LRI, O	Mint, sweet	1	38.1 ± 1.3c	36.4 ± 0.3c	113.3 ± 12.1b	133.3 ± 13.4a
15	1-Hexanol	C_6_H_14_O	1349	N.A.	MS, LRI	Flower, green	2500	N.D.	N.D.	18.9 ± 2.6	N.D.
16	1-Octen-3-ol	C_8_H_16_O	1445	981	MS, LRI, O	Mushroom	2	10.6 ± 0.3b	N.D.	16.6 ± 1.8a	14.3 ± 1.3a
17	*cis*-4-Thujanol	C_10_H_18_O	1462	1071	MS, LRI	Balsamic	N.A.	15.7 ± 0.2	N.D.	N.D.	N.D.
18	Linalool	C_10_H_18_O	1541	1101	MS, LRI, O	Aniseed, citrus	6	13.4 ± 0.7b	9.7 ± 1.8c	18.9 ± 1.5a	12.7 ± 1.3b
19	1-Octanol	C_8_H_18_O	1553	N.A.	MS, LRI, O	Herbal, green	110	N.D.	N.D.	9.2 ± 0.7	N.D.
20	Terpinen-4-ol	C_10_H_18_O	1601	1183	MS, LRI, O	Musty, terpene	340	38.4 ± 0.6b	31.4 ± 4.3bc	72.9 ± 3.9a	34.1 ± 1.0c
21	α-Terpineol	C_10_H_18_O	1695	1196	MS, LRI, O	Oil, anise, mint	350	11.1 ± 0a	5.7 ± 0.2c	N.D.	8.4 ± 0.8b
22	2-Phenylethanol	C_8_H_10_O	1908	N.A.	MS, LRI	Perfumy, rose	1100	N.D.	24.3 ± 1.2	N.D.	N.D.
	Ketones (5)							0.6 ± 0.1d	9.6 ± 1.6c	40.7 ± 4.5a	15.0 ± 1.2b
23	2-Butanone	C_4_H_8_O	885	N.A.	MS, LRI	Ethereal, cheesy	35400	N.D.	N.D.	N.D.	11.7 ± 0.9
24	2-Heptanone	C_7_H_14_O	1179	890	MS, LRI, O	Blue cheese	140	N.D.	N.D.	34.9 ± 3.6	N.D.
25	6-Methyl-5-hepten-2-one	C_8_H_14_O	N.A.	987	MS, LRI, O	Pepper, rubber	50	0.6 ± 0.1	N.D.	N.D.	N.D.
26	2-Nonanone	C_9_H_18_O	1386	N.A.	MS, LRI, O	Hot milk, green	200	N.D.	N.D.	5.9 ± 1.0	N.D.
27	Piperitone	C_10_H_16_O	1583	1260	MS, LRI, O	Mint, fresh	N.A.	N.D.	9.6 ± 1.6	N.D.	3.4 ± 0.2
	Esters (3)							47.6 ± 0.8b	92.9 ± 4.7a	16.2 ± 3.9c	10.7 ± 1.6c
28	Ethyl acetate	C_4_H_8_O_2_	869	N.A.	MS, LRI	Fruity, sweet	5	15.5 ± 0.2b	92.9 ± 4.7a	N.D.	10.7 ± 1.6c
29	Ethyl hexanoate	C_8_H_16_O_2_	1229	N.A.	MS, LRI, O	Apple, sweet	30	32.1 ± 0.6	N.D.	N.D.	N.D.
30	Terpinyl acetate	C_12_H_20_O_2_	1695	1354	MS, LRI	Fruity, mint	N.A.	N.D.	N.D.	16.2 ± 3.9	N.D.
	Hydrocarbons (14)							34.8 ± 1.8c	99.6 ± 3.2b	591.0 ± 18.4a	46.9 ± 4.7c
31	Decane	C_10_H_22_	993	1206	MS, LRI	Irritant	741	N.D.	N.D.	20.8 ± 2.4	N.D.
32	Toluene	C_7_H_8_	1031	765	MS, LRI, O	Rubber, pungent	1550	10.0 ± 1.0c	16.7 ± 1.4b	N.D.	23.9 ± 2.8a
33	β-Pinene	C_10_H_16_	N.A.	975	MS, LRI	Benzene-like	140	N.D.	N.D.	22.6 ± 0.9	N.D.
34	Ethylbenzene	C_8_H_10_	1122	N.A.	MS, LRI, O	Ethereal, floral	2205.3	N.D.	N.D.	5.0 ± 1.5	N.D.
35	*p*-Xylene	C_8_H_10_	1133	870	MS, LRI,	N.A.	490	N.D.	6.6 ± 0.2b	9.5 ± 0.4a	N.D.
36	3-Carene	C_10_H_16_	1144	1012	MS, LRI, O	Resin, lemon	0.4	N.D.	N.D.	45.2 ± 3.3	N.D.
37	α-Terpinene	C_10_H_16_	1172	1018	MS, LRI	N.A.	200	11.3 ± 0.4b	7.6 ± 1.7c	16.8 ± 3.7a	N.D.
38	Dodecane	C_12_H_26_	1076	1200	MS, LRI	Irritant	2040	N.D.	17.7 ± 0.6a	N.D.	16.4 ± 1.1a
39	d-Limonene	C_10_H_16_	1186	1031	MS, LRI, O	Fresh	10	N.D.	16.8 ± 1.9b	263.9 ± 13.6a	N.D.
40	β-Phellandrene	C_10_H_16_	1202	N.A.	MS, LRI, O	Turpentine, mint	8	N.D.	11.8 ± 2.2b	95.7 ± 6.4a	N.D.
41	*trans*-β-Ocimene	C_10_H_16_	1231	1039	MS, LRI	Sweet, herb	N.A.	N.D.	N.D.	67.5 ± 4.4	N.D.
42	*p*-Cymene	C_10_H_14_	1265	1027	MS, LRI, O	Fruity, herbal	13	12.3 ± 0.5b	11.5 ± 1.3b	36.4 ± 6.1a	6.6 ± 1.7b
43	1,3,5-Trimethylbenzene	C_9_H_12_	1277	N.A.	MS, LRI	Peculiar	229	N.D.	8.4 ± 1.8	N.D.	N.D.
44	Naphthalene	C_10_H_8_	1741	N.A.	MS, LRI, O	Camphoric	60	1.2 ± 0.1c	4.6 ± 1.0b	7.6 ± 0.8a	N.D.
	Ethers (2)							529.3 ± 24.6a	127.9 ± 3.1c	398.5 ± 2.3b	93.9 ± 6.9d
45	Estragole	C_10_H_12_O	1667	1201	MS, LRI, O	Liquorice-like	6	105.3 ± 1.6a	5.1 ± 0.4c	21.8 ± 1.5b	4.6 ± 0.6c
46	Anethol	C_10_H_12_O	1824	1290	MS, LRI, O	Aniseed-like	15	424.0 ± 22.9a	122.8 ± 3.5c	376.8 ± 3.7b	89.2 ± 6.7d
	Phenols (2)							10.6 ± 2.6c	32.1 ± 1.1b	117.8 ± 11.3a	N.D.
47	Eugenol	C_10_H_12_O_2_	2164	1364	MS, LRI, O	Spicy, clove	7.1	6.6 ± 1.5c	32.1 ± 1.1b	117.8 ± 11.3a	N.D.
48	Isoeugenol	C_10_H_12_O_2_	2255	N.A.	MS, LRI	Floral, spicy	N.A.	4.0 ± 1.1	N.D.	N.D.	N.D.
	Furans (7)							71.3 ± 2.9d	269.1 ± 13.3b	230.0 ± 13.6a	63.3 ± 2.8c
49	2-Methylfuran	C_5_H_6_O	847	N.A.	MS, LRI	Chocolate	3500	N.D.	21.0 ± 1.4a	N.D.	12.2 ± 2.5b
50	2-Ethylfuran	C_6_H_8_O	952	N.A.	MS, LRI, O	Rubber, pungent	2.3	N.D.	N.D.	11.9 ± 2.5	N.D.
51	2-Pentylfuran	C_9_H_14_O	1225	992	MS, LRI, O	Fruity, sweet	6	21.0 ± 2.7d	28.3 ± 5.8c	178.2 ± 3.7a	40.2 ± 1.2b
52	Furfural	C_5_H_4_O_2_	1457	829	MS, LRI, O	Almond, sweet	3000	26.6 ± 0.4b	62.8 ± 3.4a	16.7 ± 4.4c	5.1 ± 0.3d
53	2-Acetylfuran	C_6_H_6_O_2_	1500	912	MS, LRI, O	Sweet, smoky	80000	12.6 ± 0b	30.7 ± 1.5a	12.7 ± 2.9b	N.D.
54	5-Methylfurfural	C_6_H_6_O_2_	1568	N.A.	MS, LRI, O	Almond, sweet	1100	4.6 ± 0.4b	92.9 ± 2.5a	N.D.	N.D.
55	2-Furanmethanol	C_5_H_6_O_2_	1652	863	MS, LRI, O	Sweet, honey	2000	6.5 ± 0.2bc	33.4 ± 3.7a	11.5 ± 3.9b	5.8 ± 0.6c
	*N*-containing compounds (3)							17.8 ± 1.8a	8.5 ± 0.4b	N.D.	5.5 ± 0.3c
56	2-Methylpyrazine	C_5_H_6_N_2_	1262	N.A.	MS, LRI, O	Popcorn	250	N.D.	N.D.	N.D.	5.5 ± 0.3
57	1-Vinylimidazole	C_5_H_6_N_2_	1263	N.A.	MS, LRI	N.A.	N.A.	N.D.	8.5 ± 0.4	N.D.	N.D.
58	2-Acetylpyrazine	C_6_H_6_N_2_O	1624	N.A.	MS, LRI, O	Nutty, roast	62	17.8 ± 1.8	N.D.	N.D.	N.D.
	*S*-containing compounds (4)							12.9 ± 1.2a	4.3 ± 1.2c	4.2 ± 1.0c	7.5 ± 0.8b
59	3-Methylthiophene	C_5_H_6_S	1083	N.A.	MS, LRI, O	Fatty, wine	5000	N.D.	4.3 ± 1.2b	N.D.	7.5 ± 0.8a
60	2-Methylthiophene	C_5_H_6_S	1075	N.A.	MS, LRI, O	Gasoline, green	3000	N.D.	N.D.	4.2 ± 1.0	N.D.
61	2-Acetylthiazole	C_5_H_5_NOS	1644	N.A.	MS, LRI, O	Caramel, sweet	10	10.7 ± 1.5	N.D.	N.D.	N.D.
62	3-(Methylthio)propanol	C_4_H_10_OS	1712	N.A.	MS, LRI	Sweet, potato	123	2.2 ± 0.8	N.D.	N.D.	N.D.
	Total							1224.2 ± 34.7b	1073.4 ± 35.3c	1791.8 ± 64.8a	822.7 ± 26.2d

Note: Different letters indicate significant differences (*p* < 0.05) among samples. All experiments were conducted for *n* = 3 independent marinated pork hocks. Standard deviations are shown. MS, mass spectrum comparison using NIST libraries; LRI, linear retention index compared with literature value; O, odour description. ^e^ Odour descriptions were mainly gathered from the following literature and online database: [11,12,13,20,27,28], http://www.flavornet.org, http://www.odour.org.uk. ^f^ Odour thresholds were mainly obtained from the literature and an online database, with water applied as the matrix: [11,12,13,20,22], http://www.flavornet.org, http://www.odour.org.uk. ^g^ Linear retention index on DB-wax column. ^h^ Linear retention index on DB-5 column. ^i^ N.D., not detectable. ^g^ N.A., not available.

**Table 2:** OAVs of odour-active compounds in marinated pork hocks.

Code	Compounds	Odour Activity Values (OAVs)				*p* Value	VIP Value
		DHM	DXC	HHT	TFH		
1	2-Methylbutanal	0.0b	10.0 ± 1.2a	0.0b	0.0b	0.000	0.827
2	3-Methylbutanal	0.0b	10.6 ± 1.6a	0.0b	0.0b	0.000	0.824
3	Hexanal	20.3 ± 0.9a	3.6 ± 0.4d	11.7 ± 1.2b	8.9 ± 0.3c	0.000	0.817
4	Heptanal	8.0 ± 0.5a	8.5 ± 1.0a	0.0c	5.5 ± 0.8b	0.000	1.068
5	Octanal	47.5 ± 3.2a	30.3 ± 2.6c	35.9 ± 3.1b	45.6 ± 0.2a	0.000	0.867
6	Nonanal	166.6 ± 4.6a	122.4 ± 9.5c	68.6 ± 4.9d	135.9 ± 3.2b	0.000	1.078
7	2-Octenal isomer	1.2 ± 0.4a	0.0b	0.0b	0.0b	0.000	0.946
9	2-Nonenal isomer	22.2 ± 5.7a	0.0b	0.0b	0.0b	0.000	0.956
11	2,4-Decadienal isomer	225.5 ± 17.7a	0.0b	0.0b	0.0b	0.000	0.980
14	1,8-Cineole	38.1 ± 1.3c	36.4 ± 0.3c	113.3 ± 12.1b	133.3 ± 13.4a	0.000	0.954
15	1-Octen-3-ol	5.3 ± 0.2c	0.0d	8.3 ± 0.9a	7.2 ± 0.7b	0.000	0.943
16	Linalool	2.2 ± 0.1b	1.6 ± 0.3c	3.1 ± 0.2a	2.1 ± 0.2b	0.000	0.978
28	Ethyl acetate	3.1 ± 0b	18.6 ± 0.9a	0.0d	2.1 ± 0.3c	0.000	0.892
29	Ethyl hexanoate	1.1 ± 0.0a	0.0b	0.0b	0.0b	0.000	0.983
36	3-Carene	0.0b	0.0b	113.0 ± 8.3a	0.0b	0.000	1.078
39	d-Limonene	0.0c	1.7 ± 0.2b	26.4 ± 1.4a	0.0c	0.000	1.082
40	β-Phellandrene	0.0c	1.5 ± 0.3b	12.0 ± 0.8a	0.0c	0.000	1.081
42	*p*-Cymene	0.9 ± 0.0b	0.9 ± 0.1b	2.8 ± 0.5a	0.5 ± 0.1b	0.000	1.039
45	Estragole	17.5 ± 0.3a	0.9 ± 0.1c	3.6 ± 0.2b	0.8 ± 0.1c	0.000	0.934
46	Anethol	28.3 ± 1.5a	8.2 ± 0.2c	25.1 ± 0.2b	5.9 ± 0.4d	0.000	0.791
47	Eugenol	0.9 ± 0.2c	4.5 ± 0.2b	16.6 ± 1.6a	0.0d	0.000	1.073
50	2-Ethylfuran	0.0b	0.0b	5.2 ± 1.1a	0.0b	0.000	1.060
51	2-Pentylfuran	3.5 ± 0.5d	4.7 ± 0.1c	29.7 ± 0.6a	6.7 ± 0.2b	0.000	1.085
61	2-Acetylthiazole	1.1 ± 0.2a	0.0b	0.0b	0.0b	0.000	0.974

Different letters refer to statistically significant differences (*p* < 0.05). Values represent means and standard deviation (*n* = 3).

**Table 3:** Ingredient composition of different marinades based on the product labels.

Products	Ingredients
DHM	Pork hock, salt, soy sauce, white granulated sugar, flavour liquor, soy protein, monosodium glutamate.
DXC	Pork hock, salt, soy sauce, white granulated sugar, glucose, rice wine, soy protein, spices, monosodium glutamate, pork seasoning.
HHT	Pork hock, salt, soy sauce, white granulated sugar, glucose, soy protein, spices.
TFH	Pork hock, salt, sugar, baijiu, spices.

**Table 4:** Definitions of odour attributes and reference standards.

Odour Attributes	Definitions	References (Intensity)
Fatty	The smell associated with lard oil	Lard oil at 25 °C (6.0)
Meaty	The smell associated with cooked pork	20.0 g of defatted pork in 60.0 mL of water was boiled for 1 h (8.0)
Soy sauce	The smell associated with soy sauce	3.0 g of soy sauce in 50.0 mL of water (7.0)
Fruity	The smell associated with fresh fruit	Newly cut orange peel or apple peel (8.0)
Caramel	The smell associated with burning white sugar	5.0 g of burning white sugar in 50.0 mL water (6.0)
Roasted	The smell associated with roasted pork	1.0 kg pork was roasted by charcoal fires for 1 h (6.0)
